# Irisin, Fibroplast Growth Factor-21, and Follistatin Responses to Endurance Rowing Training Session in Female Rowers

**DOI:** 10.3389/fphys.2021.689696

**Published:** 2021-06-04

**Authors:** Jaak Jürimäe, Sille Vaiksaar, Priit Purge, Vallo Tillmann

**Affiliations:** ^1^Institute of Sport Sciences and Physiotherapy, Faculty of Medicine, University of Tartu, Tartu, Estonia; ^2^Institute of Clinical Medicine, Faculty of Medicine, University of Tartu, Tartu, Estonia

**Keywords:** myokines, metabolism, aerobic capacity, training stress, female athletes

## Abstract

**Purpose:** This study examined selected myokine responses to an endurance rowing training session, and whether metabolic demands of the acute aerobic rowing exercise together with training volume, aerobic capacity, and body composition variables affect potential exercise-induced changes in the myokine levels in female rowers.

**Methods:** Fifteen national level female rowers [18.3 ± 1.6 years; 172.0 ± 5.0 cm, 67.5 ± 8.8 kg; maximal oxygen consumption (VO_2_max): 47.2 ± 7.9 ml.min.^−1^ kg^−1^] performed a 1-h rowing ergometer exercise at the intensity of 70% of VO_2_max [distance: 12.1 ± 1.1 km; energy expenditure (EE): 639 ± 69 kcal; heart rate (HR): 151 ± 7 beats.min^−1^] followed by a 30-min recovery period. Venous blood samples were collected before and after exercise, and analyzed for irisin, fibroplast growth factor-21 (FGF-21), and follistatin conentrations.

**Results:** Plasma irisin and FGF-21 concentrations were increased (by 8%; *p* = 0.013 and by 13%; *p* < 0.0001, respectively) immediately after the aerobic rowing exercise. Follistatin was significantly increased (by 11%; *p* = 0.001) only after the first 30 min of recovery. Exercise metabolic demand variables such as distance covered and total EE were correlated with the pre-to-post-exercise increases in FGF-21 concentrations (*r* = 0.52; *p* = 0.047 and *r* = 0.68; *p* = 0.005, respectively). Exercise-induced increases in irisin levels were related to aerobic capacity as measured by VO_2_max (*r* = 0.53; *p* = 0.041) and training stress as measured by weekly training volume (*r* = 0.54; *p* = 0.039) in female rowers.

**Conclusion:** Acute negative energy balance induced by a single endurance rowing training session elicited significant increases in irisin, FGF-21, and follistatin levels in national level female rowers. While exercise-induced increases in FGF-21 levels were associated with exercise metabolic demand measures, exercise-induced increases in irisin concentrations were related to aerobic capacity and training stress measures in female rowers.

## Introduction

Rowing training is mainly focused on improving aerobic capacity (Rämson et al., 2008; Jürimäe and Purge, 2021), and an increase in prolonged low-intensity endurance rowing training sessions has been observed during the last decades (Fiskerstrand and Seiler, 2004; Jürimäe and Purge, 2021). It appears that endurance training below anaerobic threshold (AnT) is the mainstay of success in rowing (Fiskerstrand and Seiler, 2004; Mäestu et al., 2005). However, in response to high-volume low-intensity rowing training sessions, some athletes may not be able to maintain sufficient energy intake (Rämson et al., 2008; Kurgan et al., 2018), which can lead to a negative energy homeostasis in these athletes (Jürimäe et al., 2011). The regulation of negative energy homeostasis and high training stress is also dependent on several peripheral factors that communicate the status of body energy stores to the brain (Jürimäe and Jürimäe, 2005; Jürimäe et al., 2011). These peripheral factors are also synthesized from adipose, muscle, and bone tissues, which may act as endocrine organs (Kirk et al., 2020). Accordingly, a recent study used such peripheral markers as tumor necrosis factor-alpha, interleukin-6 (IL-6), leptin, and insulin-like growth factor-1 to assess variations in energy homeostasis and training stress over a training year in elite female rowers (Kurgan et al., 2018). In addition, acute exercise-induced negative energy balance may also contribute to the regulation of these peripheral markers of energy homeostasis (Jürimäe and Jürimäe, 2005; Jürimäe et al., 2011). While adipose tissue produces different adipokines that are involved in the regulation of energy metabolism (Jürimäe et al., 2011), different myokines have also been reported to play an important role in energy metabolism during acute exercise (He et al., 2018; Kirk et al., 2020).

Various myokines have been suggested to mediate exercise-induced energy expenditure (EE; He et al., 2018, 2019), besides the most investigated and well known myokine, IL-6 (Rämson et al., 2008; Ives et al., 2011; Jürimäe et al., 2011; Kirk et al., 2020). These myokines include myostatin (Sliwicka et al., 2021), follistatin (Perakakis et al., 2018), irisin (Qiu et al., 2018), and fibroplast growth factor-21 (FGF-21; Larsen et al., 2020) that have recently emerged as potential mediators of exercise-induced energy metabolism in physically active individuals. Myostatin, a member of the transforming growth factor β family of cytokines and the first described peripheral signal from muscle tissue to fulfill the criteria of a myokine (He et al., 2018), is a negative regulator of muscle mass (Kirk et al., 2020). In contrast, follistatin is a myostatin-binding peptide that promotes skeletal muscle development through the activation of anabolic pathways (He et al., 2019). Myostatin and follistatin also exert metabolic benefits by reducing body fat mass (FM), browing of white adipose tissue (WAT) and improving glucose homeostasis (Braga et al., 2014; He et al., 2019). In addition, irisin is one of the more newly identified myokine, primarily secreted by muscle tissue and released into the circulation during exercise, resulting in an increased EE, reduced body FM and improved glucose metabolism (Boström et al., 2012). FGF-21 has also been proposed as a myokine with metabolic effects on glucose and lipid metabolism, and promoting body FM loss and WAT browning (Kirk et al., 2020; Khalafi et al., 2021). Recent studies have demonstrated that acute aerobic exercise sessions with different duration could be positively associated with increased circulating irisin (Qiu et al., 2018; Sliwicka et al., 2021), FGF-21 (Larsen et al., 2020; Khalafi et al., 2021), and follistatin (Perakakis et al., 2018; He et al., 2019) concentrations in individuals with different physical activity levels. However, it has also been suggested that acute exercise sessions represent potential influence for the myokine releases only when they are characterized by adequate intensity and/or stimuli of exercise (Fatouros, 2018; He et al., 2019). Furthermore, the amount of muscle mass involved during acute exercise may contribute to the post-exercise myokine release (Hansen et al., 2011; Qiu et al., 2018). To our best knowledge, no studies have yet investigated the effect of acute prolonged rowing exercise session on circulating irisin, FGF-21, and follistatin concentrations in female athletes. Accordingly, rowing is an exercise mode where all major muscle groups are involved and this type of exercise protocol produces greater energy stimulus (Rämson et al., 2008) that might be needed for exercise-induced myokine release in comparison with running or cycling exercises.

The purpose of the present investigation was to evaluate the effects of prolonged aerobic rowing training session on circulating irisin, FGF-21, and follistatin concentrations in female rowers. Another aim was to examine whether metabolic demand values of the acute rowing exercise together with training stress, aerobic capacity, and body composition variables affect potential exercise-induced changes in the myokine levels in female rowers. It was hypothesized that irisin, FGF-21, and follistatin concentrations will increase as a result of acute negative energy balance caused by aerobic rowing exercise, and that the increases in some myokine concentrations will be associated with metabolic demand measures of acute rowing exercise in female rowers.

## Materials and Methods

### Participants

Fifteen national level female rowers with a rowing training experience of 5.1 ± 1.8 years participated in this study (Table 1). This investigation involves a further analysis of blood samples previously collected and analyzed, and inflammatory cytokine responses to endurance exercise have been previously reported in these female rowers (Jürimäe et al., 2018). All participants were eumenorrheic female rowers with a menstrual cycle duration of 24–35 days, and were not using oral contraceptive pills for at least 6 months preceding the study (Dean et al., 2003; Vaiksaar et al., 2011a). They were asked to document their menstrual cycles for at least 6 months together with the measurement of body mass on a weekly basis (Dean et al., 2003). To be included in the study, rowers had to be weight stable for the last 6 months (Dean et al., 2003), and have a body composition of more than 12% body fat to rule out potential endogeneous hypothalamic-gonadal endocrine axis dysfunction (Ives et al., 2011). Accordingly, the criterion to be weight-stable was that the body mass change was less than 3 kg during the 6-month training period (Dean et al., 2003). The study was conducted during the preparatory period for the competitive rowing season, where the main goal of training was to increase the aerobic base through extensive aerobic training sessions (Rämson et al., 2008). The mean training volume of studied female rowers was 7.3 ± 3.2 h.week^−1^, and the training intensity was below AnT for approximately 90% of the entire training time (Rämson et al., 2008). All female rowers were fully familirized with the purpose, procedures, and possible risks of the study before providing their written consent to participate. None of the participants had any significant health problems or were taking any medication before the study period. The experimental protocol was conducted in accordance with the Declaration of Helsinki and was approved by the Medical Ethics Committee of the University of Tartu, Estonia.

**Table 1:** Mean (±SD) subject characteristics in female rowers.

Variable	Mean ± SD
Age (years)	18.3 ± 1.6
Height (cm)	172.0 ± 6.0
Body mass (kg)	67.5 ± 8.8
Body fat %	28.4 ± 5.0
Fat mass (kg)	18.6 ± 5.1
Fat free mass (kg)	46.7 ± 5.8
Muscle mass (kg)	16.8 ± 2.5
AnT (W)	174 ± 30
HR at AnT (beats.min^−1^)	172 ± 7
HRmax (beats.min^−1^)	189 ± 6
Pmax (W)	241 ± 36
VO_2_max (l.min^−1^)	3.2 ± 0.6
VO_2_max/kg (ml.min.^−1^ kg^−1^)	47.2 ± 7.9
Estradiol (pmol.l^−1^)	170.1 ± 75.1
Progesterone (nmol.l^−1^)	1.6 ± 0.6

AnT, anaerobic threshold; HR, heart rate; Pmax, maximal aerobic power; and VO_2_max, maximal oxygen consumption.

### Experimental Design

All participants completed one preliminary session followed by one experimental session during the follicular phase of the menstrual cycle (determined as days 7–11 from onset of menstruation, mean day 9 ± 2 for the main experimental session; Suh et al., 2002; Vaiksaar et al., 2011a). Menstrual cycle phase was later confirmed by estradiol and progesterone concentrations from the blood samples (Suh et al., 2002; Vaiksaar et al., 2011a,b). Preliminary testing included incremental rowing ergometer test, which was performed between 4:00 and 6:00 p.m. followed by body composition measurement. Main experimental testing consisted of an 1-h endurance rowing ergometer session that was conducted on the following day after the incremental rowing ergometer test between 4:00 and 6:00 p.m. (Vaiksaar et al., 2011b). Rowers were in a post-absorptive state having eaten a meal about 2 h before both exercise tests. Athletes were asked to maintain their usual daily dietary habits and everyday activities before testing. In addition, participants abstained from any dietary supplementation before the first visit to the laboratory (Vaiksaar et al., 2011a,b).

### Measurements

#### Body Composition

The height (Martin metal anthropometer, GMP Anthropological Instruments, Zurich, Switzerland) and body mass (A&D Instruments Ltd., Oxfordshire, United Kingdom) of the rowers were measured to the nearest 0.1 cm and 0.05 kg, respectively. Body composition was measured *via* dual-energy X-ray absorptiometry using Lunar DPX-IQ Densitometer (Lunar Corporation, Madison, WI, United States) and analyzed for total body fat percent, FM, and fat free mass (FFM). In addition, lower-leg skeletal muscle mass (muscle mass) was calculated as the sum of legs’ lean soft-tissue masses (Jürimäe et al., 2018). The coefficient of variation (CV) for body composition measurements was less than 2%.

#### Incremental Rowing Ergometer Test

Stepwise incremental rowing ergometer test was performed on a wind resistance-braked rowing ergometer (Concept II, Morrisville, VT, United States) to determine maximal oxygen consumption (VO_2_max) and also target heart rate (HR) indices for a 1-h endurance exercise protocol (Jürimäe et al., 2001; Vaiksaar et al., 2011b). Participants were equipped with the instruments and sat quietly for 1 min on the rowing ergometer before starting to exercise at 40 W for 1 min. Workload was increased by 15 W after every minute until volitional exhaustion (Vaiksaar et al., 2011a,b). HR was recorded every 5 s during the test using Sporttester Polar 725X (Polar Electro Oy, Kempele, Finland). Respiratory gas exchange variables were measured throughout the test in a breath-by-breath mode using portable open circuit spirometry system (MetaMax 3B, Cortex Biophysik GmbH, Germany) and data were stored in 10 s intervals (Hofmann et al., 2007). Oxygen consumption (VO_2_), carbon dioxide production (VCO_2_), minute ventilation (V_E_), breathing frequency (f_B_), and tidal volume (V_T_) were continuously measured. The mean respiratory exchange ratio (RER) and ventilatory equivalents of O_2_ (V_E_/VO_2_) and CO_2_ (V_E_/VCO_2_) were calculated from the recorded measurements (Hofmann et al., 2007). All data were processed by means of computer analysis using a standard software (MetaSoft; Cortex Biophysik GmbH, Germany) and VO_2_max was obtained as described previously (Hofmann et al., 2007). Specifically, to determine that VO_2_max was reached, attainment of the VO_2_ plateau with increasing work rate was used as a criterion. However, when the VO_2_ plateau was not observed, an RER exceeding 1.1 and a theoretical maximal cardiac frequency were used as a criteria. The test was designed to reach the maximum in approximately 15 min in each participant (Hofmann et al., 2007). AnT determination was performed using linear regression turnpoint analysis, which has been found to be reliable in determining the individual intensity for aerobic-anaerobic transition in rowers (Hofmann et al., 2007).

#### Main Experimental Session

The exercise session consisted of rowing on a rowing ergometer for 1 h at the intensity of 70% VO_2_max (Jürimäe et al., 2001; Vaiksaar et al., 2011b). At first, rowers rested quietly for 10 min in a seated position, and baseline (PRE) blood samples were obtained. Blood samples were also collected immediately after the rowing session (POST) and after the first 30 min of the recovery period (POST-30). Target HR was set at the level obtained from the incremental test using a practical set ±2 beats.min^−1^ of 70% VO_2_max (Jürimäe et al., 2001; Vaiksaar et al., 2011b). Participants were instructed to increase exercise intensity smoothly and the requested HR was achieved after the first 5 min. The exercise intensity at the requested HR corresponded to RER < 1 (Vaiksaar et al., 2011b). The rowers were instructed to maintain the target HR steady state for the entire exercise session and to reduce exercise intensity to accommodate the required HR steady state as needed (Jürimäe et al., 2001; Vaiksaar et al., 2011b). Respiratory gas exchange variables were measured throughout the 1-h rowing ergometer session in a breath-by-breath mode using a portable open circuit spirometry system (MetaMax 3B, Cortex Biophysik GmbH, Germany) as described above. Exercise EE was estimated from the RER using stoichiometric equations (Frayn, 1983), with the assumption that urinary nitrogen excretion rate was neglible (Venables et al., 2005). These equations have previously been used in females to assess submaximal exercise EE, during which the RER was <1 (Suh et al., 2002; Venables et al., 2005). In addition, rating of perceived exertion (RPE, 6–20 scale) was assessed (Borg, 1970) and capillary blood samples for enzymatic determination of blood lactate (Lange, Germany) were collected before and after the 1-h rowing ergometer exercise (Vaiksaar et al., 2011b).

#### Blood Analysis

A 10-ml blood sample was obtained, and blood plasma was separated and frozen at −20°C for subsequent analysis. All blood samples from the same individual were analyzed at the same time. Irisin was determined using ELISA kit using specific Irisin/FDNC5 monoclonal antibody (R&D Systems Inc., Minneapolis, MN, United States). This assay had intra‐ and inter-assay CVs 2.5 and 8.7%, respectively, and the least detection limit was 0.25 ng.ml^−1^. FGF-21 was assessed by a commercially available ELISA kit (R&D Systems Inc., Minneapolis, MN, United States) with a minimum detectable level of 1.61 pg.ml^−1^, and intra-assay CV 3.5% and inter-assay CV 5.2%. Follistatin was measured using commercially available ELISA kit (R&D Systems Inc., Minneapolis, MN, United States) with a minimum detectable level of 29 pg.ml^−1^, and intra-assay CV 3.0% and inter-assay CV 10.0%. In addition, estradiol and progesterone were determined on Immulite 2000 (DPC, Los Angeles, CA, United States). The intra‐ and inter-assay CVs for estradiol were 5.3 and 6.5%, and for progesterone 5.4 and 3.4%, respectively. The routine complete blood counts were performed by our clinical hematology laboratory, and provided blood hemoglobin and hematocrit values. The CVs for all hematological variables were less than 2%. Plasma volume changes were estimated according to the method of Dill and Costill (1974).

### Statistical Analysis

Statistical analyses were performed using SPSS software version 21.0 package for Windows (Chicago, IL, United States). Data are presented as mean ± SD. Evaluation of normality was performed with Shapiro-Wilks method. Data that were not normally distributed were logarithmically transformed prior to analyses to approximate normal distribution (Jürimäe et al., 2018). One-way ANOVA and least significant difference *post hoc* analysis tests were used to evaluate differences between time points (Vaiksaar et al., 2011b). In addition, effect size (ES) transformations were used to compare the magnitude of change in myokine values as a consequence of the aerobic rowing ergometer session. Differences between mean values of each measured myokine contrasting pre‐ to post-exercise levels were transformed to ESs (Rhea, 2004). ESs approximating <0.25, 0.25–0.49, 0.50–1.0, and >1.0 were categorized as trivial, small, moderate, and large changes, respectively (Rhea, 2004). Spearman correlations were used to evaluate bivariate relationships among different variables of interest (Jürimäe and Jürimäe, 2005). The level of significance was set at *p* < 0.05.

## Results

Plasma estradiol and progesterone concentrations confirmed the follicular phase of the menstrual cycle in female rowers (Table 1). The average HR at AnT (172 ± 7 beats.min^−1^) corresponded to 91.0 ± 2.6% of their HRmax (189 ± 6 beats.min^−1^) and AnT was achieved at 83.4 ± 6.7% of VO_2_max (2.64 ± 0.44 l.min^−1^). In the main experimental session, female athletes rowed over a distance of 12.1 ± 1.1 km, with a mean exercise HR of 151 ± 7 beats.min^−1^ or 79.6 ± 4.5% of the HRmax. Total EE of the 1-h aerobic rowing exercise trial was 639 ± 69 kcal with a mean EE rate of 10.6 ± 1.2 kcal.min^−1^. Body mass was reduced (*p* < 0.0001) after the exercise trial from 67.5 ± 8.8 to 66.3 ± 8.9 kg. The mean RPE as an exercise intensity measure during the aerobic exercise was 12.6 ± 1.2. Before exercise, blood lactate concentration was 1.9 ± 0.6 mmol.l^−1^, which did not change significantly as a result of the exercise trial (2.3 ± 1.0 mmol.l^−1^). Changes in plasma volume as a result of the aerobic rowing exercise were small (−0.4 ± 1.7%).

**Figure 1:**
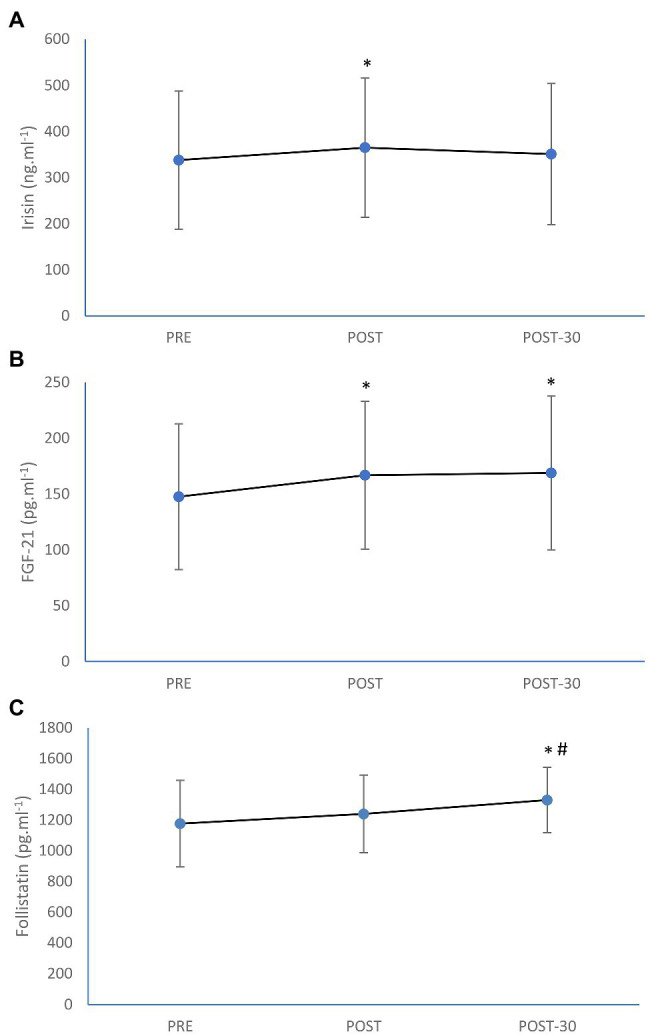
Mean (±SD) irisin **(A)**, fibroplast growth factor-21 (FGF-21; **B**), and follistatin **(C)** concentrations before (PRE), immediately after (POST), and after 30-min rest (POST-30) of 1-h of prolonged rowing exercise in female rowers. ^*^Significantly different from PRE; *p* < 0.05. ^#^Significantly different from POST; *p* < 0.05.

Circulating irisin concentration was significantly increased (by 8%; *p* = 0.013) as a result of rowing exercise trial (Figure 1). Significant increment immediately after the exercise as well as after 30 min of recovery was also seen in FGF-21 level (by 13%; *p* < 0.0001 and by 14%; *p* < 0.0001, respectively), while post-exercise increase in follistatin (by 4%) was not significant (*p* = 0.065). However, follistatin was significantly increased after the first 30 min of recovery (by 11%; *p* = 0.001) in comparison with the pre-exercise value (Figure 1). The magnitude of increase in irisin was trivial (ES = 0.18), while the magnitude of increases in FGF-21 (ES = 0.28) and follistatin (ES = 0.31) were small as a result of aerobic rowing exercise.

From the metabolic demand measurements of the 1-h aerobic rowing ergometer exercise trial, the mean distance covered (*r* = 0.52; *p* = 0.047) and total EE (*r* = 0.68; *p* = 0.005) values were significantly correlated with the pre-to-post-exercise increase in FGF-21 concentration. Metabolic demand measurements of 1-h aerobic rowing ergometer exercise were not associated with the pre-to-post-exercise increases in other measured myokine values (*r* < 0.36; *p* > 0.05). In addition, significant correlations of the pre-to-post-exercise increases in irisin level with VO_2_max (*r* = 0.53; *p* = 0.041) and weekly training volume (*r* = 0.54; *p* = 0.039) were observed. Age, aerobic capacity, body composition including muscle mass, sex hormone, and training volume variables were not further related to exercise-induced increases in any measured myokine concentrations (*r* < 0.47; *p* > 0.05).

## Discussion

The present study assessed the responses of specific myokines to an aerobic rowing exercise session that could be involved in metabolic regulation of the exercise stress in female athletes. Therefore, the used prolonged rowing exercise provided a valid reflection of sport-specific endurance capacity in female rowers (Jürimäe et al., 2001, 2021). Specifically, the intensity of the prolonged rowing exercise corresponded to the intensity of aerobic threshold as measured by blood lactate concentrations, which did not change significantly during the exercise trial (before: 1.9 ± 0.6 mmol.l^−1^; after: 2.3 ± 1.0 mmol.l^−1^). In addition, although the level of exercise intensity did not cause any significant changes in plasma volume, all measured plasma myokine values were corrected for exercise-induced plasma volume changes similarly to recent relevant acute exercise studies (Kouvelioti et al., 2019; Larsen et al., 2020; Jürimäe et al., 2021). Accordingly, the post-exercise increases in measured myokine concentrations in female rowers appear to be truly induced by exercise as also reported by previous acute exercise studies with non-athletic male participants (He et al., 2018, 2019). Furthermore, exercise-induced increases in FGF-21 levels were related to the amount of metabolic reaction as indicated by the total EE of the rowing exercise (Vaiksaar et al., 2011b). In contrast, the measured exercise intensity values such as the mean RPE and HR of the rowing exercise (Nieman et al., 2012) were not related to the pre-to-post-exercise changes in any measured myokine values. The results of the present study demonstrate that irisin, FGF-21, and follistatin could be regarded as signals for metabolic reaction for the energy requirements during and after aerobic rowing exercise, while FGF-21 levels can be used as an indicator for the amount of energy metabolism in female rowers.

In the current study, significant increase (by 8%; *p* = 0.013) in circulating irisin level immediately after the aerobic rowing exercise was observed, while the increase was trivial (ES = 0.18) in magnitude in female rowers. Only few studies have investigated the response of irisin to acute exercise in athletes, and increases (Kabasakalis et al., 2020), no change (Vassalle et al., 2020; Joro et al., 2021) or even decreases (Sanderson et al., 2020) in irisin concentrations as a result of acute exercise have been observed. In contrast to our results, circulating irisin levels have been reported to be more sensitive to exercise intensity that is known to be an important stimulator for irisin release (Fatouros, 2018; Kabasakalis et al., 2020). It has been argued that for circulating irisin concentration to increase, the acute exercise parameters should induce relatively high blood lactate production in athletes (Kabasakalis et al., 2020). The biological role of irisin as a moderator of energy metabolism in response to acute exercise still remains not fully elucidated (Fatouros, 2018; Sliwicka et al., 2021), although irisin is considered to be a factor regulating muscle adaptation, including stimulation of glucose uptake and lipid metabolism (Arhire et al., 2019; Lee and Jun, 2019; Jaworska et al., 2021). Typically, previous studies have reported increases in irisin concentrations immediately after different acute exercises with the effect being independent of the mode of exercise session (i.e., running or cycling) in healthy men (Daskalopoulou et al., 2014; Huh et al., 2014; Fatouros, 2018; Qiu et al., 2018), and a meta-analysis showed that the mean magnitude of post-exercise increase in irisin concentration across studies is around 15% in non-athletic individuals (Fox et al., 2017). However, there are also recent studies demonstrating no acute exercise effects and no differences between conditions (i.e., moderate intensity continuous training, sprint interval training, high-intensity interval training, or resistance training) for circulating irisin levels in healthy non-athletic participants (He et al., 2018, 2019; Reycraft et al., 2020). It has also been argued that irisin may be a marker of muscle damage and act as a protective agent (Vaughan et al., 2015), and also that irisin may provide an anti-inflammatory protection in fat cells as a result of acute exercise (Fatouros, 2018). In addition, our findings suggest that the level of aerobic capacity and weekly training stress of female rowers may influence post-exercise irisin release. Specifically, acute exercise-induced increases in plasma irisin concentrations were related to VO_2_max (*r* = 0.53; *p* = 0.041) and weekly training volume (*r* = 0.54; *p* = 0.039) in female rowers. Similarly, Fox et al. (2017) suggested that fitness level might be the major predictor of exercise-induced irisin release, while other studies have observed that exercise-induced irisin secretion is independent of fitness level (Huh et al., 2014; Qiu et al., 2018). However, the subjects in these studies were recreationally active male individuals (Huh et al., 2014; Qiu et al., 2018) and not athletes, as were the studied participants in our study. In addition, muscle mass and sex hormone values were not associated with pre-to-post-exercise increases in irisin concentrations in studied female rowers. In accordance, different stages of menstrual cycle did not influence irisin release in response to 90 min of aerobic running exercise in recreationally trained women with normal menstrual cycle (Kraemer et al., 2014). Taken together, the results of our acute rowing exercise session suggest that a not yet exactly defined exercise stimulus is needed for a post-exercise increase in irisin concentration, and post-exercise increase in irisin level may also depend on the physical condition of the studied female rowers. However, there is lack of data in athletes to make a definitive conclusion about the role of irisin in energy metabolism of acute exercise.

The present investigation showed that a 1-h aerobic rowing ergometer exercise with an EE of 639 ± 69 kcal over a distance of 12.1 ± 1.1 km increased plasma FGF-21 concentration by 13% (*p* < 0.0001) although the magnitude of increase was small (ES = 0.28). Recently, circulating FGF-21 concentration was increased immediately after a marathon race in male recreational marathon runners (Larsen et al., 2020), while another study observed no increases in FGF-21 levels immediately after 30-min of running exercise at the intensities of 50 and 80% of VO_2_max in healthy male volunteers (Kim et al., 2013). However, significant increases in FGF-21 concentrations were found 1-h post-exercise in both exercise intensity conditions, while the increase in post-exercise FGF-21 level was significantly higher after exercise with higher intensity (i.e., 80% of VO_2_max) in comparison with the corresponding value after exercise with lower intensity (i.e., 50% of VO_2_max; Kim et al., 2013). Accordingly, a dose-response relationship between plasma FGF-21 and exercise intensity was suggested (Kim et al., 2013). In addition, He et al. (2019) assessed the response of circulating FGF-21 concentration to increasing running exercise loads, and found that the amount of exercise load modified the FGF-21 response to acute exercise in healthy untrained men. Specifically, higher exercise load at the intensity of AnT (total EE of 762 ± 118 kcal.h^−1^) produced significantly higher post-exercise FGF-21 response in comparison with a lower exercise load at the intensity of maximum fat oxidation rate (total EE of 480 ± 160 kcal.h^−1^; He et al., 2019). In accordance, our female rowers demonstrated that the pre-to-post-exercise increase in plasma FGF-21 level was related to metabolic demand measurements including the mean distance covered (*r* = 0.52; *p* = 0.047) and total EE (*r* = 0.68; *p* = 0.005) of the performed rowing exercise. Based on the role of FGF-21 on glucose uptake and lipolysis (Kim et al., 2013; Khalafi et al., 2021), it is suggested that exercise-induced increased FGF-21 level mediates beneficial effects of acute exercise on glucose and lipid metabolism. In addition, to our best knowledge, this is the first study in athletes, which showed that circulating FGF-21 level was significantly elevated as a result of acute endurance exercise and that exercise-induced EE as an indicator of acute exercise stress was significantly associated with pre-to-post-exercise increases in FGF-21 level. Accordingly, circulating FGF-21 concentration could be used as a marker of energy homeostasis and exercise stress in female rowers. However, this issue needs further investigations in athletes before any conclusions may be drawn.

Follistatin has been reported to bind myostatin in order to inactivate it, and consequently promote skeletal muscle development (Hansen et al., 2011; Perakakis et al., 2018). Interestingly, He et al. (2018) reported that the increase in myostatin concentration upon acute exercise termination occurred concomitantly with an increase in follistatin only 3-h after acute high-intensity interval treadmill running exercise in non-athletic men. Similarly, 3-h bicycle exercise at the intensity of 50% of VO_2_max did not cause an immediate post-exercise increase in circulating follistatin concentration, while plasma follistatin increased during the recovery period peaking 3-h after exercise in healthy untrained males (Hansen et al., 2011). In the present study, circulating follistatin concentration was significantly increased only after the first 30-min of recovery from the 1-h aerobic rowing exercise in female rowers. Therefore, the increase in follistatin magnitude was small (ES = 0.31). In another study, follistatin level was increased immediately after 36 min of moderate-intensity run on treadmill in healthy untrained men and continued to increase 1 h after recovery period (Perakakis et al., 2018). In accordance, because follistatin concentration was significantly higher after the first 30 min of recovery period in comparison with the corresponding value obtained immediately after the rowing exercise, we cannot exclude a further increase in follistatin concentration over a longer period in our studied female rowers. Moderate increases in serum follistatin levels have been observed to occur for few days after the eccentric exercise-induced muscle damage in healthy men (Philippou et al., 2017). It has been suggested that post-exercise increases in follistatin levels stimulate the energy turnover in skeletal muscle to meet the energy demands during recovery from exercise (Philippou et al., 2017; Perakakis et al., 2018), while the exercise-induced increase in follistatin may also be dependent on the muscle mass recruited during the exercise bout (Hansen et al., 2011). Taken together, the specific exercise mode and protocol, where about 70% of all body muscles are involved in rowing (Rämson et al., 2008), provided a sufficient stimulus to increase plasma follistatin concentration already after the first 30-min of recovery period of the acute rowing exercise in studied female rowers. However, further studies with athletes are needed to better understand the suitability of using circulating follistatin concentration as a myokine marker to characterize acute exercise stress in trained athletes.

The main strength of the investigation is the study population consisting of trained female athletes, who make up a rather homogeneous group of subjects in terms of specific aerobic performance, body composition, and also sex hormone variables. However, it has to be considered that the results of the present study cannot be extrapolated to other populations such as sedentary individuals, people with obesity and aging individuals. Another strength of the investigation is that, to the best of our knowledge, this was the first study that measured irisin, FGF-21, and follistatin responses to exercise simultaneously in female athletes and used direct measurement of oxygen consumption in order to assess the metabolic demands of the aerobic rowing exercise. The main limitations of the present investigation are the observational study design with no control trial and the absence of the longer and more frequent sampling period during recovery after exercise trial. In addition, the lack of measurement of some important myokines such as myostatin is another limitation of the present study. Accordingly, more research is needed to analyze the effect of acute training loads measuring myokines together with specific adipokines and osteokines with a longer recovery period to better characterize acute exercise stress and negative energy balance in female athletes. Finally, another research area should focus on studying these myokines as markers of chronic exercise stress and possible overtraining in various athletic populations.

In summary, our study demonstrated that circulating plasma irisin and FGF-21 concentrations were increased immediately after the prolonged endurance rowing training session, while follistatin levels were elevated after the first 30-min of recovery period in female rowers. Metabolic demand values (covered distance and EE) of the rowing exercise session were related to the exercise-induced increases in circulating FGF-21 levels, whereas aerobic capacity as measured by VO_2_max and training stress as measured by weekly training volume were correlated with exercise-induced increases in irisin concentrations. These results suggest that plasma irisin, FGF-21, and follistatin levels could be used as biological markers of acute exercise stress in female rowers.

## Data Availability Statement

The raw data supporting the conclusions of this article will be made available by the authors, without undue reservation.

## Ethics Statement

The studies involving human participants were reviewed and approved by Medical Ethics Committee of the University of Tartu. The patients/participants provided their written informed consent to participate in this study.

## Author Contributions

JJ, SV, PP, and VT designed the study. SV and PP performed the research. JJ wrote the manuscript. SV, PP, and VT reviewed and edited the manuscript. All authors contributed to the article and approved the submitted version.

### Conflict of Interest

The authors declare that the research was conducted in the absence of any commercial or financial relationships that could be construed as a potential conflict of interest.
